# An Adaptive Healthcare Organization Can Effectively Respond to Medical Crises

**DOI:** 10.3389/ijph.2023.1605581

**Published:** 2023-08-11

**Authors:** Jocelyn Carter, Harry Burke

**Affiliations:** ^1^ Massachusetts General Hospital and Harvard Medical School, Boston, MA, United States; ^2^ Department of Medicine, F. Edward Hébert School of Medicine, Uniformed Services University of the Health Sciences, Bethesda, MD, United States

**Keywords:** adaptive healthcare organization, coronavirus, pandemic, medical crisis, COVID

## Abstract

Healthcare systems are challenged by unexpected medical crises. Established frameworks and approaches to guide healthcare institutions during these crises are limited in their effectiveness. We propose an Adaptive Healthcare Organization (AHO) system as a framework focused on the dynamic nature of healthcare delivery. Based on seven key capabilities, the AHO framework can guide single and multi-institutional healthcare organizations to adapt in real time to an unexpected medical crisis and improve their efficiency and effectiveness.

## Adaptive Health Care Organizations

The SARS-CoV-2 pandemic overwhelmed healthcare organizations all over the world. A qualitative study examining the experiences of clinical, administrative, and custodial staff working in hospitals and nursing homes in Sweden during the COVID-19 pandemic [1] reported that the medical crisis was characterized by four themes. These themes were identified as 1) inconsistent and unclear communication, 2) suboptimal leadership at various levels, 3) insufficient resources and equipment, and 4) overwhelming workloads leading to high burnout. Descriptions of clinical staff isolation, anxiety, helplessness, and concerns over the lack of preparedness of the pandemic have also been well-described [2] and are consistent with the experiences of those in healthcare writ large. While retrospective review has demonstrated clear gaps in preparedness for the COVID-19 pandemic, here we seek to 1) explore the underlying etiological factors contributing to the ineffective response of healthcare organizations and 2) propose a novel approach to healthcare crisis strategy. This new approach is based on adaptive principles that will enable healthcare organizations to improve responsiveness to medical crises in the future.

While frontline healthcare organizations often have well prepared plans and algorithms for recurring disasters like floods, fires, airplane crashes, and explosions, they are generally unprepared for a crisis like an anthrax attack or a novel coronavirus pathogen [3]. A medical crises, by definition, is an event that challenges the frontline of a medial organization and requires a medical response beyond the scope of a healthcare organization’s established activities or protocols. Specific dimensions of a medical crises include: size (local, national, or worldwide), intensity (affects a few, a moderate number, or many people), and duration (acute, continuing, or chronic) [4]. Additional aspects of medical crises are onset (sudden or gradual emergence) and degree of anticipation (known and expected like a hurricane; known and unexpected like a plane crash; or unknown and unexpected like an anthrax). The COVID-19 pandemic was a perfect storm with a global, intense, chronic, sudden, unknown and unexpected appearance.

When healthcare organizations are challenged with unpredictable threats, a number of historical and more recent strategies have been invoked. One traditional approach to healthcare challenges focuses on the 4Ss of preparedness: staff, stuff, space, and systems [5]. But, as Barbash et al point out [6], many hospitals struggled with COVID-19 surges despite lead time and extensive resources to implement the 4S framework. Instead, Barbash et al. advocate for healthcare system resilience characterized by four key elements: 1) well-staffed workforces with appropriate training, skills, and experience, 2) preserved standards of care delivery integrating protocoled clinical pathways, 3) preserved access to care with optimized electronic health records, and 4) protection of the wellbeing of frontline personnel by fostering a supportive work environment. While this approach has a strong foundation, it deepens reliance on what may be interpreted as optimal pre-existing capabilities of a healthcare organization to solve future threats. This concept is idealized at best and sets a very high baseline standard of functionality for organizations. In reality, this standard may not be an attainable baseline for many healthcare institutions.

The learning healthcare organization (LHO) model is another way a system could respond to a healthcare crisis [7]. Its mission is to “capture data from clinical encounters and other health-related events, analyze the data to generate new knowledge, and then apply this knowledge to continuously inform and improve health decision-making and practice.” [8] This principle can add value in a number of healthcare settings. However, because learning healthcare organizations are generally focused on longitudinal evidence-based improvements, the utility of this approach in a true crisis can be limited. LHO activities are often methodological and take time—time that institutions responding to an immediate, existential medical crisis do not have.

The high reliability organization (HRO) model has been one of the most well-described and longest-standing organizational approaches. It relies on five key principles: 1) sensitivity to operations, 2) reluctance to simplify, 3) preoccupation with failure, 4) deference to experts, and 5) practicing resilience [9]. A high reliability organization is defined as one that experiences fewer than anticipated accidents or harmful events of harm while operating in a highly complex, high-risk environment. It was initially created in response to critical events in industry (e.g., a nuclear power plant hazard or an airplane crash) to minimize internal operating failures. The HRO model has since been adapted to healthcare settings in an effort to avoid operational hazards. This approach is often applied in a way that is deviation-focused with primary attention on detecting and correcting operating errors. This model, while helpful in specific quality assurance and improvement endeavors, has a restrictive scope that does not capture the time-sensitive core capabilities required for healthcare organizations to respond to an external threat like a pandemic.

When facing a new medical crisis, frontline healthcare organizations must be ready to effectively respond to the challenge in real time. To meet this challenge, we propose the Adaptive Healthcare Organization (AHO). This model emphasizes proactive, innovative, and collaborative strategy built upon a foundation of organizational flexibility and rapid decision-making based on the experiences of frontline healthcare workers. The goal of an AHO is not to return to previously established best practice. Rather, the AHO goal is to reorganize itself to effectively respond to present and future conditions that may be entirely unfamiliar. As a new way of looking at organizational structure and function, the AHO model also emphasizes the dynamic nature of operations faced during a healthcare crisis. An AHO has the following seven core capabilities (Table 1).

**TABLE 1:** Principles of adaptive healthcare organizations (Adaptive Healthcare Organization, USA, 2023).

**Open lines of communication within and across functional units**	
*Global Aim:* Encourages messaging of clear goals, objectives as well as pending tasks	● Prioritization of transparency by both leaders and the workforce
*Logic:* Clear communication can enhance understanding of barriers, and facilitators and driving more actionable and impactful planning	● Development of dedicated pathways to relay important updates and emerging issues
**Ability to capture, analyze, and act on information in real time**	
*Global Aim:* Emphasize reporting of data metrics with balanced process and outcome measures designed for hour-to hour, day-to-day or week-to-week review	● Rooted in high-quality information transfer to generate well-informed strategy decisions
*Logic:* Incorporation of metrics of measure that capture operations in real time can inform what is working and what is not working	● Relies on real time data reporting without unnecessary delays
**Possess a flexible organizational structure**	
*Global Aim:* Leadership openness to pilot top ideas generated with workforce guidance	● Leadership reliance and connection to frontline staff
*Logic:* Application of strategies driven by inclusive strategic planning from the frontline can lead to more efficient and effective outcomes	● Action teams to assist with implementation
	● Changes made based on frontline worker feedback
	● Cross-training of staff
**Ability to innovate, try new methods, and learn quickly from mistakes**	
*Global Aim:* Emphasis on lessons learned from pilots with application to future scenarios; includes timely evaluation of any changes (fail fast mentality)	● Focus on frontline worker observations
	● Cross-training of staff
*Logic:* Strategy and decision making informed by real-time experience can lead to better solutions with fewer delays	
**Ability to incorporate anticipatory and proactive measures**	
*Global Aim:* Promotion of real-time feedback as evidence base for strategic planning	● Real time modeling and simulation prioritizes day-to-day and week-to-week needs until further reserves for emerging challenges can be established
*Logic:* Use of hourly and daily metrics can highlight current and potential emerging issues	● Strategic design of the planning process should reflect anticipated need for future changes as time moves forward
**Maintain sufficient personnel, supplies, and resources to effectively respond to the crisis**	
*Global Aim:* Focus on pre-crises allocation of funding for resources to support early phases of crises	● Movement away from pure reliance on just-in time supplies
	● Incorporation of pre-planning for primary resources and allocation of funding for this as a pre-investment
*Logic:* Promotion of more comprehensive investment in resources prior to crises can help avoid disruption and enhance operations during crises	● Help avoid supply-demand mismatch price surging during crises
**Respect for personnel at all levels**	
*Global Aim:* Emphasis on frontline worker sustainability and optimization	● Cross-training of staff
	● Leadership effort in frontline roles
	● Providing mental services to frontline clinicians creates a standard and culture that elevates workforce needs
*Logic:* Early normalization of mental health and work life balance resources can promote efficiency and effectiveness	● Simulating reasonable crisis-mode working hours that reflect user-centered design reduces burn-out and dissatisfaction

## Capture, Analyze, and Act on Information in Real Time

Databases must be immediately available to receive, store, and assemble crisis-related information (eliminating the need for *ad hoc* spreadsheets). As events unfold, critical information must be transmitted in real time and accessible to the relevant staff and patients. Experts must be available to implement algorithms and statistical methods to model the data and provide frontline clinicians with the information needed to make efficient, effective, and safe medical decisions. There must be continuous feedback of outcomes to the frontline clinicians. This was described by Hoffman and associates in the creation of a systems to track telehealth use via session outputs [10].

## Innovate, Try New Methods, and Learn Quickly From Mistakes

Preplanned responses to previous disasters should not be immediately implemented. Information about the crisis should be gathered in real time and responses should be tailored to the current situation. Furthermore, a particular solution may not work everywhere in the organization because of local functions and conditions. Multiple solutions should be piloted with only those demonstrating improvement being implemented. In addition, key workforce staff should be cross-trained so that critical functional roles can be quickly reassigned. Konda et al. emphasized this in their description of developing a hybrid orthopedic surgeon-internal medicine service based on treatment pathways [11].

## Incorporate Anticipatory and Proactive Measures

Organizations must take a proactive approach to problems in order to avoid operations becoming entrenched and inflexible. With the assistance of frontline staff to provide immediate feedback, the identification of emerging issues as they develop can inform proactive strategies and the execution of anticipatory initiatives. This was exhibited in Chaudhury et al.’s description of how rapid and robust feedback from frontline on COVID- test result delays necessitated the creation of in-house rapid- testing as a solution to a clogged clinical workflow [12].

## Possess a Flexible Organizational Structure

One of the most ineffective approaches to medical crises is top-down decision-making, where the leadership of an organization makes the critical decisions. This is ineffective not only because are there too many decisions for leadership to make but also because leadership usually lacks the ability to tailor decisions to local issues in a knowledgeable and timely manner. The more effective approach is to assemble action teams composed of frontline clinical staff, relevant medical domain experts, medical crisis specialists, and administrators. These teams can consider changes proposed by frontline staff (and leadership) and help guide leadership’s decision-making and vetting process. In other words, actions should be based on a bidirectional process where the experiences of frontline staff are valued as highly as (or higher than) the expertise of leaders and the lessons learned can assist frontline personnel in their activities. Konda et al. underlines this in their description of how a failed strategy to deploy clinicians to the ED was quickly abandoned in favor of more strategic utilization of nurse practitioners as advocated by the work force.

## Maintain Open Lines of Communication Within and Across Functional Units

Communication tends to deteriorate during a medical crisis as information systems become overwhelmed. In truth, the current technologies and modes of communication, both horizontal and vertical, routinely used by healthcare organizations are inadequate to meet the demands of an unexpected medical crisis. Organizations should add communication modalities, channels, equipment, software, and protocols that allow them to communicate with all employees and patients in real time to provide up-to-the-minute status information. Channels should allow for clinicians to perform virtual health visits with patients and directly communicate with one another within and across functional units. Communication between staff should be immediate and accurate; and it should target specific levels, units, and roles. Alvarez et al emphasized how challenged healthcare leaders were to stay in close communication with frontline workers during the pandemic while implementing virtual care and other operational changes [13].

## Respect Personnel at All Levels

During a medical crisis, clinical staff are asked to work long hours under dangerous conditions and perhaps even outside their scope of practice. These circumstances can heighten stress and lead to burnout. Initiatives like cross-training as well as having leadership spend time on the front lines can aid in preserving and promoting dignity, respect, and understanding of the roles and experiences of frontline staff. Financial rewards should also be provided as tangible recognition of workforce hard work, dedication, and sacrifices. In addition, social services and wellness resources should be provided at the onset to assist frontline staff in dealing with the disruption of their personal lives caused by the crisis. There were few health systems that were prepared to address the level of distress experienced by frontlines during the pandemic. However, Svantesson et al captured staff needs for additional support well in their study identifying high levels of stress and anxiety experienced by 52% and 40% of respondents, respectively in a cross-sectional survey of 1,074 healthcare professionals during the pandemic [14].

## Maintain Sufficient Personnel, Supplies, and Resources to Effectively Respond to the Crisis

Many organizations believe in being “lean,” which often can translate to having the fewest possible employees, the least supplies, and as little money as possible available for emergencies. Unfortunately, when a medical crisis occurs, this positioning can greatly restrict an organization’s responsiveness when tackling an unexpected threat. In fact, responding effectively to a medical crisis requires adequate personnel, equipment, and monetary resources. Medical organizations must have contingency funds in their budgets in order to meet the initial demands of a medical crisis. Organizations should not rely on pure just-in-time supply chains for critical equipment and supplies or promote sustained minimalist staffing ratios. While governments were eventually able to find ways to reduce the supply-demand mismatch as the COVID-19 pandemic unfolded, these methods were delayed, unconventional, and could have been avoided with better pre-crisis planning [15].

We acknowledge that many healthcare organizations face economic, staffing, and other resource-related constraints. Such limitations can make integrating our adaptive strategies more challenging. Furthermore, the historical and cultural epicenter of healthcare institutions is one that values stability and often equates it with remaining operationally static or fixed over time. Our AHO model emphasizes flexibility and being open to system improvement. Consequently, the AHO approach can not only guide an organization during crises, but it can prompt an examination of the current state and priorities of a healthcare organization prior to a crisis. We believe that this kind of organizational self-examination can not only inform which aspects of the AHO may be actionable when a crisis arises but can also function as an essential and necessary step toward any healthcare organization’s ability to adapt in its routine environment. In this way, the AHO approach can promote evaluation and, in doing so, has the potential to improve the long-term efficiency and effectiveness of healthcare organizations. This is a unique characteristic of the AHO strategy that sets it apart from other crisis related strategies.

In conclusion, the SARS-CoV-2 pandemic created an unexpected medical crisis that challenged healthcare organizations and revealed significant weaknesses in healthcare systems around the world. It is a wake-up call to 21st century medicine that we must evolve from static systems that attempt to maintain current function during crises to dynamic systems, ever changing and adapting, and able to successfully meet the next medical challenge. By adopting the Adaptive Healthcare Organization model, healthcare systems can ensure a more robust and effective response to future unexpected medical crises.
